# Treatment of Delirium Tremens by Large Doses of Digitalis

**Published:** 1860-10

**Authors:** 


### ARTICLE XXII.

### *Treatment of Delirium Tremens by large Doses of Digitalis.*

In the number of September 29th of the Medical Times and Gazette, Dr. G. M. Jones, Surgeon to the Jersey General Hospital, reports his treatment of Delirium Tremens by large doses of Digitalis. As the subject is of considerable interest, showing how large doses of this drug may be borne in certain conditions, we give Dr. Jones' views somewhat in full.

"About twelve years ago I was called to see a patient with delirium tremens, residing about a mile from my house, who was almost in articulo. I prescribed a dose of chloric ether with tincture of opium; but the wife, who came for the medicine, took, by mistake, a phial containing one ounce of digitalis. I discovered the error, and was horrified when I heard that the patient had taken this dose; but no less surprised than pleased when I also heard that, instead of being poisoned, he was very much better. Under ordinary treatment I fully believed he would have died; but after this singular dose he rapidly recovered. Profiting by this hint, I began to give digitalis in all cases of delirium tremens which came under my care in hospital and private practice, and during the last twelve years, I have adopted it in at least seventy cases—this effect of drunkenness being very common in Jersey.

"As to the dose, experience has taught me that the best dose is half an ounce of the tincture given in a little water. In some few cases, this one dose is enough, but generally a second dose is required four hours after the first. In some cases, but very seldom, a third dose is called for, but this hardly ever need exceed two drachms. The largest quantity I have ever given was half an ounce at first, half an ounce four hours afterward, and another half ounce six hours after that—making an ounce and a half in ten hours.

As to the effects of these doses, my impression is that the action is on the brain, not on the heart. The pulse so far from being lowered in force, becomes fuller and stronger and more regular, soon after the first dose. The cold clammy perspirations pass off, and the skin becomes warmer. As soon as the remedy produces its full effect, sleep for five or six, or seven hours commonly follows; sleep is the guide as to the repetition of the dose. No action on the kidneys is evidenced by any unusual secretion of the urine. Sometimes the bowels are slightly acted on, but not commonly. I have never once seen any alarming symptom follow the use of these large doses of digitalis. The only case I have lost since adopting this treatment, had a tumor in the brain. In three only was other treatment adopted after digitalis had failed to procure sleep; in other words, in sixty-seven out of seventy cases, digitalis was the only medicine used, and sixty-six of these patients recovered. I do not mean that these are the exact numbers of those treated; I am certain as to the death, but I may have had more recoveries. I am well within bounds in saying seventy cases in twelve years, and that all of them were well-marked cases of delirium tremens. Slight cases of nervous derangement after drinking I have seen in great numbers, but I speak here only of such cases as required active treatment. My previous experience of the results of the treatment by opium, or some of its preparations, by anti-spasmodics, etc., had certainly been much less successful; the proportion of deaths was much larger, and the recovery much less rapid. Again, I have treated more than one patient successfully by digitalis, who in subsequent attacks elsewhere, has been treated by opium and died; and in many of the cases in which I have used digitalis successfully, opium had been previously given without any good effect.

